# Stumpfe Gewalt in der forensischen Radiologie

**DOI:** 10.1007/s00117-024-01366-1

**Published:** 2024-09-25

**Authors:** Wolf-Dieter Zech, Thomas D. Ruder

**Affiliations:** 1 Institut für Rechtsmedizin Bern, Universität Bern, Murtenstrasse 26, 3008 Bern, Schweiz; 2 Universitätsinstitut für Diagnostische, Interventionelle und pädiatrische Radiologie, Inselspital, Universitätsspital Bern, Universität Bern, Rosenbühlgasse 27, 3010 Bern, Schweiz

**Keywords:** Nichtakzidentelle und akzidentelle Verletzungen, Häusliche Gewalt, Rechtsmedizin, Postmortale Computertomographie, Postmortale Magnetresonanztomographie, Nonaccidental and accidental injuries, Domestic violence, Forensic medicine, Postmortem computed tomography, Postmortem magnetic resonance imaging

## Abstract

**Hintergrund:**

Darstellung der wesentlichen forensisch-radiologischen Untersuchungsmodalitäten und Befunde bei stumpfer Gewalt bei lebenden und verstorbenen Erwachsenen.

**Methoden:**

Herausarbeitung der wesentlichen Punkte anhand eigener praktischer Erfahrung der Autoren sowie einschlägiger Fachliteratur.

**Ergebnisse und Schlussfolgerung:**

Verletzungsbedingte Folgen stumpfer Gewalt sind in der forensisch-radiologischen Diagnostik häufig zu beobachten, vor allem im Rahmen von Unfällen und Suiziden, seltener bei Tötungsdelikten. Die Methode der Wahl zur radiologischen Darstellung von stumpfer Gewalt bei Verstorbenen ist die native postmortale Computertomographie (PMCT). Grundsätzlich unterscheiden sich Folgen stumpfer Gewalt radiologisch in der PMCT nicht wesentlich vom Lebenden. Die postmortale Magnetresonanztomographie (PMMR) ist im kürzeren postmortalen Intervall sehr gut zur Darstellung von stumpfen Weichteilverletzungen geeignet. Bei Lebenden mit Folgen stumpfer Gewalt erfolgt die Indikation zur Bildgebung primär aus klinisch-diagnostischen Gründen. Häufige Indikationen sind häusliche Gewalt, Gewalt an älteren Personen sowie Auseinandersetzungen im öffentlichen Raum. Die Wahl der radiologischen Untersuchungsmethode ist abhängig von klinischer Anamnese und Symptomatik, wobei die radiologischen Untersuchungen einer forensischen Begutachtung unterzogen werden können.

## Abstract (en)

**Objective:**

Description of the main forensic radiological examination modalities and findings in blunt force trauma in living and deceased adults.

**Methods:**

Elaboration of the essential points based on the authors’ own experiences and relevant literature.

**Results and conclusion:**

Injury-related consequences of blunt force are frequently observed in forensic radiological diagnostics, especially in the context of accidents and suicides, and less frequently in homicides. The method of choice for radiological imaging of blunt force in deceased persons is native postmortem computed tomography (PMCT). In principle, the radiological effects of blunt force in PMCT do not differ significantly from those in living persons. Postmortem magnetic resonance imaging (PMMRI) is very suitable for imaging blunt soft tissue injuries in the shorter postmortem interval. In the case of living individuals with the consequences of blunt force trauma, imaging is primarily indicated for clinical diagnostic reasons. Common indications are domestic violence, violence against the elderly, and disputes in public spaces. The choice of radiological examination method depends on the clinical history and symptoms, and the radiological examinations can be subjected to a forensic assessment.

Stumpfe Gewalt wird als mechanische Einwirkung einer mehr oder minder großen Fläche auf den Körper bzw. einzelne Körperregionen definiert. Im forensischen Kontext sind als Entstehungsmechanismen stumpfer Gewalt beispielsweise Faustschläge, Fußtritte, Schläge mit stumpfen Gegenständen, Stürze (auch aus großer Höhe) oder Verkehrsunfälle zu benennen. Verletzungen und verletzungsbedingte Folgen stumpfer Gewalt gehören in der forensisch-radiologischen Diagnostik dabei mit zu den am häufigsten zu beobachtenden Befunden und ergänzen hinsichtlich der forensischen Fallbeurteilung die rechtsmedizinisch erhobenen Befunde.

## Allgemeine Aspekte und Untersuchungsmodalitäten

**Abb. 1:**
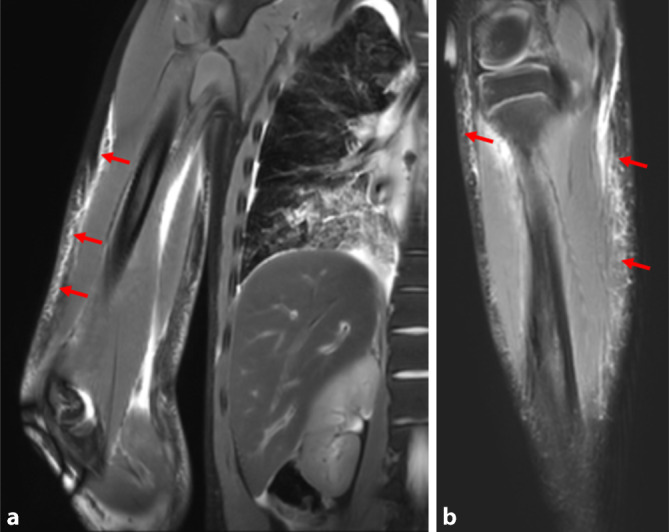
Postmortale Magnetresonanztomographie (PMMR) in T2w mit ausgedehnten Hautunterblutungen (*rote Pfeile*) an rechtem Oberarm (**a**) und rechtem Unterschenkel (**b**) bei tödlichem Verkehrsunfall als Fahrradfahrer und Sturz auf die rechte Körperseite

Stumpfe Gewalt führt, sobald sie ein gewisses Maß überschreitet, entweder durch mechanische Zug- oder Schubspannung sowie Torsions- oder Scherkräfte zu Hautverletzungen, Gefäßzerreißungen, Knochenbrüchen sowie Weichteil- bzw. Organverletzungen [4, 16, 19]. Die Methode der Wahl zur radiologischen Darstellung von direkten und indirekten Folgen stumpfer Gewalt bei verstorbenen Personen ist die native postmortale Computertomographie (PMCT) des gesamten Körpers [5, 11, 14, 15, 22, 24]. In konkreten Einzelfällen kann zur Suche eines beschädigten Gefäßes als Blutungsquelle auch eine postmortale CT-Angiographie sinnvoll sein [9]. Ein großer Vorteil det Ganzkörper-PMCT liegt darin, stumpfe Verletzungen auch in den Körperregionen aufzeigen zu können, die autoptisch nicht oder nur unter bestimmten Umständen eröffnet werden würden. Dies betrifft vor allem den Gesichtsschädel, die Extremitäten und das Rückenmark. Zur postmortalen Darstellung von stumpfen Weichteilverletzungen ist grundsätzlich auch die postmortale Magnetresonanztomographie (PMMR) sehr gut geeignet (Abb. 1), wobei dies in der Praxis, hauptsächlich aufgrund des eingeschränkten Zugangs zu entsprechenden Geräten und den im Vergleich zur PMCT deutlich höheren Kosten, aktuell nur selten angewandt wird [21].

Bei lebenden Personen mit stumpfmechanisch verursachten Verletzungen erfolgt bei Erwachsenen die Indikation zu Bildgebung primär aus klinisch-diagnostischen, und nicht aus forensisch-dokumentationsorientierten Gründen. Die Wahl der radiologischen Untersuchungsmethode ist dabei abhängig von klinischer Anamnese und Symptomatik. Die jeweils angefertigten radiologischen Untersuchungen können aber einer forensischen Begutachtung unterzogen werden. Der Hauptteil dieses Artikels bezieht sich auf die postmortale forensische Radiologie. Der letzte Abschnitt beschäftigt sich mit klinischer forensischer Radiologie bei lebenden Personen.

## Stumpfe Gewalt bei Verstorbenen

### Stumpfe Hautverletzungen

**Abb. 2:**
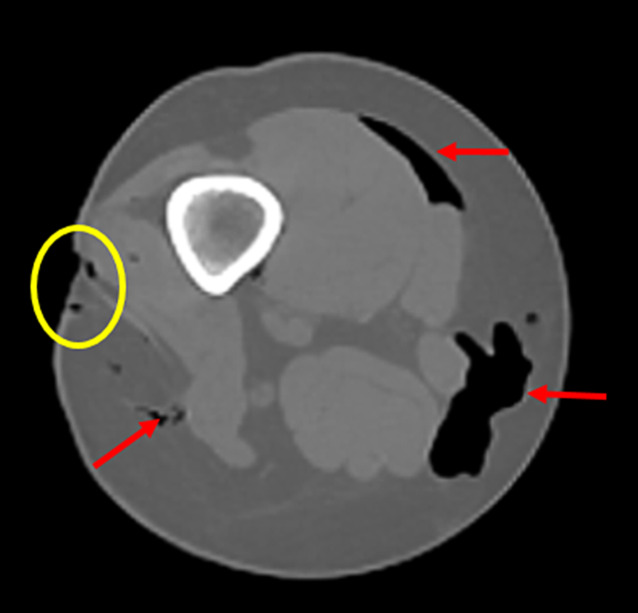
Postmortale Computertomographie (PMCT), Gas im subkutanen Weichteilgewebe (*rote Pfeile*) bei offener Hautverletzung (*gelbe Umkreisung*) am Oberschenkel nach Kollision als Fahrradfahrerin mit Auto

Hautverletzungen als Folge stumpfer Gewalt, etwa Hautabschürfungen, Hauteinblutungen, Hautunterblutungen oder Quetsch-Risswunden sind für die rechtsmedizinische Fallbeurteilung häufig von erheblicher Relevanz, da aus ihnen teils Rückschlüsse auf forensisch relevante Aspekte wie bspw. Verletzungsalter, Art des Entstehungsmechanismus, Art des einwirkenden Gegenstandes oder Richtung der Gewalteinwirkung gezogen werden können [4, 16, 19]. Insbesondere rein intrakutane Verletzungen, wie oberflächliche Hautabschürfungen und Hauteinblutungen, sind mittels PMCT oder PMMR in der Regel nicht direkt zu erkennen, was in der Praxis jedoch unerheblich ist, da diese Verletzungen ohnehin bei der äußeren rechtsmedizinischen Untersuchung akribisch erfasst und dokumentiert werden. Von forensisch-radiologischer Bedeutung, etwa bei Fragen der Vitalität einer Verletzung (d. h. ob eine Verletzung zu Lebzeiten verursacht worden ist) oder der Schwere einer Gewalteinwirkung, kann mittels PMCT oder PMMR die Ausdehnung einer stumpfmechanischen Verletzung über die Haut hinaus ins subkutane Fettgewebe und tiefere Weichteilschichten dargestellt werden [5, 15, 21, 24]. Durchtrennungen bzw. Defekte der Haut als Folge von schwereren stumpfen Gewalteinwirkungen können, wenn sie groß genug sind, in der PMCT oder PMMR erkannt werden und sind häufig von einem subkutanen Emphysem begleitet ([14, 22]; Abb. 2). Wenn derartige Verletzungen sich nur auf den Hautmantel beschränken, kann die forensisch relevante Unterscheidung, ob es sich um Folgen stumpfer oder scharfer Gewalt handelte, radiologisch jedoch schwierig bis unmöglich sein.

**Abb. 3:**
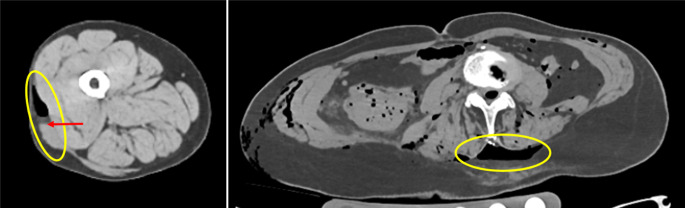
Postmortale Computertomographie (PMCT), Decollement an Rücken und rechtem Oberschenkel mit sichtbaren Taschenbildungen (*gelbe Umkreisungen*) und Ansammlung von sedimentiertem Blut (*Pfeil*) in der Tasche am Oberschenkel bei Zustand nach Überrollung

Von forensischer Relevanz sind zudem sog. Decollementverletzungen. Dabei handelt es sich um Ablederungsverletzungen, die durch tangential einwirkende stumpfe Gewalt entstehen und Hinweise auf den Entstehungsmechanismus (wie bspw. eine Überrollung durch Fahrzeugreifen) geben können. Bei derartigen Verletzungen werden durch Scherkräfte die oberen Hautschichten von den tieferliegenden Muskelfaszien und/oder dem Fettgewebe getrennt [4, 16, 19]. Dadurch entstehen Wundtaschen, die in der PMCT oder PMMR teils direkt, teils indirekt (durch eine Ansammlung von Blut in der Wundtasche) sichtbar sein können ([3, 13]; Abb. 3).

### Stumpfe Schädel-Hirn-Traumata

Schwere stumpfe Gewalteinwirkungen gegen den Kopf sind häufig von forensischer Relevanz. Beispiele dafür sind Faustschläge, Schläge mit stumpfen Gegenständen, Stürze sowie Beschleunigungsunfälle (Verkehrs- und Flugunfälle). Derartige Einwirkungen können Schädelfrakturen, Kompressionen sowie Translations- und Rotationsbeschleunigungen des Gehirns verursachen, was zu Gefäßrupturen, zerebralen Kontusionen und intrakraniellen (epiduralen, subduralen, subarachnoidalen und intraparenchymatösen) Blutungen führen kann [4, 16, 19]. *Diese Verletzungen sind mittels PMCT oder PMMR sehr gut darstellbar und vom radiologischen Befund her vergleichbar mit Schädel-Hirn-Trauma-Befunden bei Lebenden* [27]. Zur optimalen Beurteilung des Kopfes wird zusätzlich zum Ganzkörper-PMCT mit maximalem Field-of-View (FoV) eine dedizierte PMCT von Schädel und Halswirbelsäule mit angepasstem FoV durchgeführt.

**Abb. 4:**
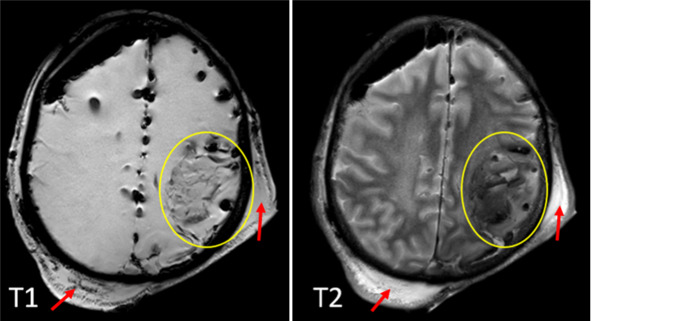
Todesursächliche linkshemisphärische intraparenchymale Hirnblutung (*gelbe Umkreisungen*) mit Kopfhautunterblutungen (*rote Pfeile*) in der postmortalen Magnetresonanztomographie (PMMR) T1w und T2w nach mehrfachen Stürzen auf den Kopf unter Blutverdünnung

Forensische Fragestellungen treten häufig bei unklarem oder strittigem Hergang eines stumpfen Schädel-Hirn-Traumas auf. In solchen Fällen ist es Aufgabe der Rechtsmedizin, den eigentlichen Entstehungshergang anhand der postmortal radiologisch und autoptisch erhobenen Befunde zu rekonstruieren [4, 16, 19]. Forensisch relevante radiologische Befunde umfassen verschiedene Schädelfrakturarten sowie Vorhandensein, Alter und Entität von intrakraniellen Blutungen sowie Coup-Contrecoup-Verletzungen [5, 11, 14, 15, 22, 24, 27]. Bei größeren Blutungen mit raumfordernder Komponente oder Blutungen im Bereich des Hirnstamms ist, abhängig von den übrigen Befunden am Körper, grundsätzlich eine todesursächliche Relevanz in Betracht zu ziehen [4, 16, 19, 26, 27]. Aus radiologischer Sicht stellen sich intrakranielle Blutungen in der PMCT, zumindest im kürzeren postmortalen Intervall, in der Regel nicht anders dar als beim Lebenden. Dieser Grundsatz gilt auch für die PMMR (Abb. 4), wobei zu beachten ist, dass das Signalverhalten verschiedener Sequenzen der PMMR bei niedriger Körpertemperatur vom Signalverhalten bei Lebenden abweichen kann [21].

Coup-Contrecoup-Verletzungen entstehen typischerweise bei einem Sturz auf den Kopf. Sie können von Verletzungen abgegrenzt werden, die als direkte Folge eines Schlages gegen den Kopf entstehen. Beim Coup-Contrecoup zeigen sich Verletzungen an der primären Anprallstelle (Coup) und an der gegenüberliegenden Hirnseite (Contrecoup). Die Contrecoup-Verletzung entsteht durch den Unterdruck, der durch die plötzliche Beschleunigung des Kopfes beim sturzbedingten Kopfanprall verursacht wird [4, 16, 19]. Radiologisch finden sich beim Coup ein Galeahämatom, die Ursprungsstelle der Schädelfraktur und zerebrale Kontusionsherde mit Blutungskomponenten. Beim Contrecoup finden sich ebenfalls Kontusionsherde, oft begleitet von subduralen Blutungen. Das Ausmaß der traumatischen Befunde ist beim Contrecoup häufig größer als beim Coup. Bei einem Sturz auf den Hinterkopf können durch Verformung des Schädels auch Frakturen im Bereich der vorderen Schädelbasis auftreten, etwa an den Orbitadächern [4, 24, 27].

**Abb. 5:**
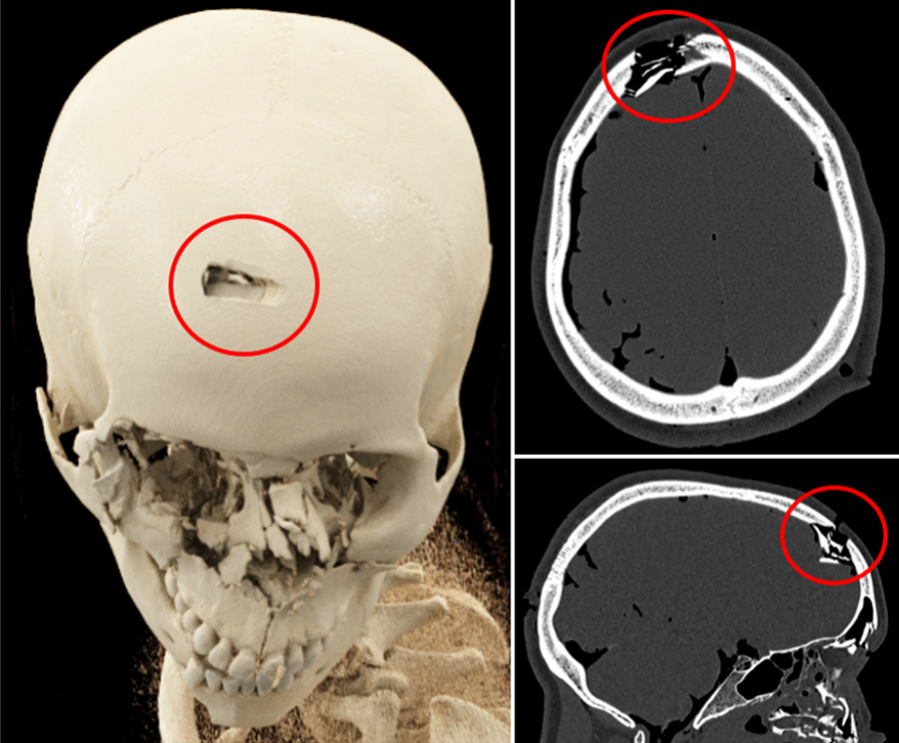
Postmortale Computertomographie (PMCT), Lochbruch bzw. Impressionsfraktur (*rote Umkreisungen*) am Stirnbein bei Tötungsdelikt mit Hammerschlägen gegen den Kopf

**Abb. 6:**
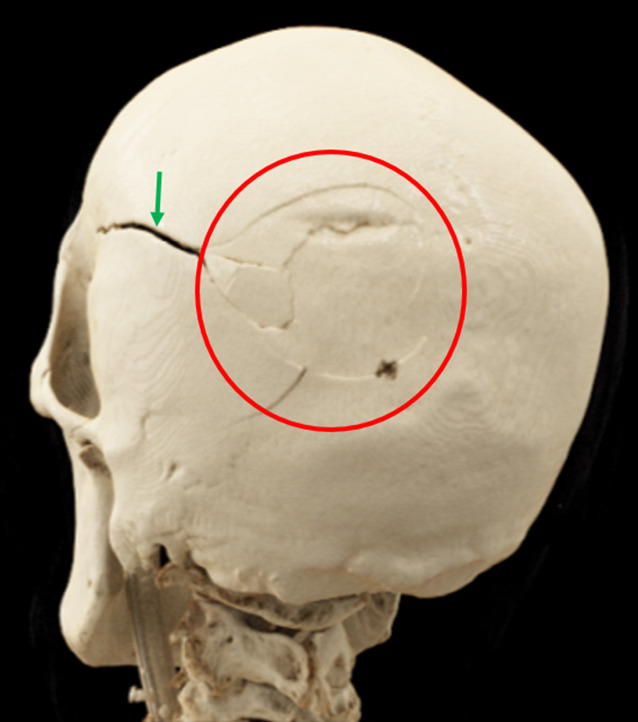
Postmortale Computertomographie (PMCT), cinematographische 3D-Rekonstruktion eines Globusbruchs mit Biegungsbrüchen (*rote Umkreisung*) und Berstungsbruch (*grüner Pfeil*) infolge Sturz auf den Hinterkopf

**Abb. 7:**
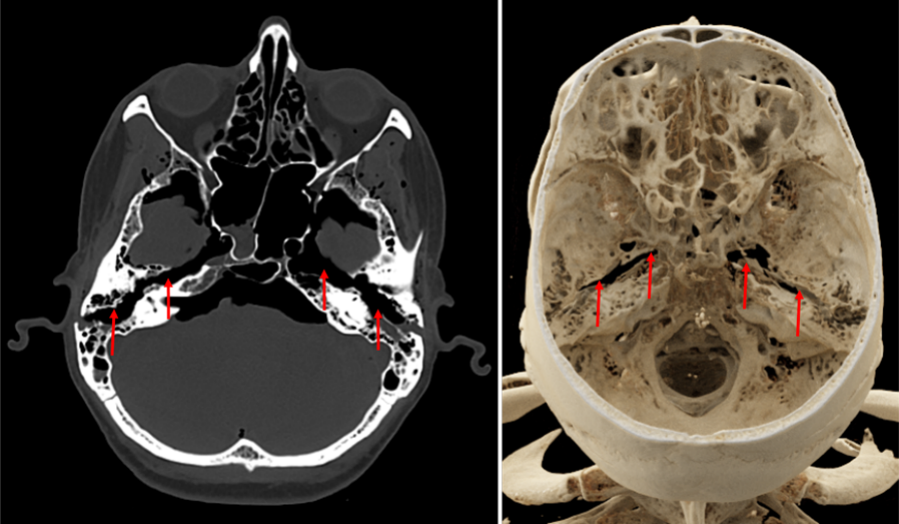
Postmortale Computertomographie (PMCT), Scharnierbruch (*rote Pfeile*) der Schädelbasis bei Zustand nach Überrollung des Kopfes durch Autoreifen

Je nach Art der stumpfen Gewalteinwirkung können Schädelfrakturen als Biegungsbrüche (direkt am Ort der Gewaltenwirkung), Berstungsbrüche (indirekt durch Verformung des Schädels infolge Zugspannung) oder Impressionsbrüche (z. B. Lochbruch infolge Hammerschlags; Abb. 5) auftreten. Weitere forensisch relevante Bruchsysteme sind bspw. Globusbrüche (Biegungsbruch mit davon ausgehenden Berstungsbrüchen bei schweren Gewalteinwirkungen; Abb. 6), Scharnierbrüche der Schädelbasis (bei beidseitiger stumpfer Gewalteinwirkung, z. B. Überrollungen des Kopfes; Abb. 7) sowie seltene Ringbrüche der Schädelbasis (bei Stauchung des Kopfes gegen die Wirbelsäule oder Zug des Kopfes an der Wirbelsäule, z. B. bei Motorradunfällen; [4, 16, 19]).

**Abb. 8:**
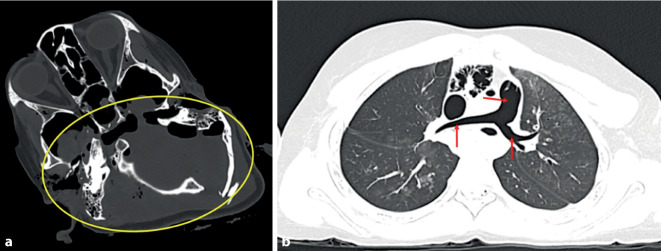
Postmortale Computertomographie (PMCT). Schwerstes Schädel-Hirn-Trauma an Hinterkopf und Schädelbasis (**a**,* gelbe Umkreisung*) infolge Sturz aus der Höhe mit ausgedehnter, todesursächlich konkurrierender Gasembolie in Truncus pulmonalis und Pulmonalarterien (**b**,* rote Pfeile*)

Bei Vorliegen von gröberen Schädelbasisfrakturen sollte radiologisch auch auf größere Blutansammlungen bzw. blutisodenses Material in der Trachea und den großen Bronchien geachtet werden, da es in diesen Fällen zu relevanten Blutungen in den Rachenraum mit todesursächlich relevanter Blutaspiration kommen kann. Offene stumpfe Schädel-Hirn-Traumata oder Schädelbasisfrakturen mit Beteiligung des Felsenbeins können zu tödlichen Luftembolien führen, die autoptisch schwer zu erkennen, aber in der PMCT sehr gut darstellbar sind ([5, 11, 15, 24]; Abb. 8). Sowohl Luftembolie als auch die tiefe Blutaspiration gelten als Vitalzeichen, weisen also darauf hin, dass eine Schädelverletzung zu Lebzeiten erfolgt sein muss. Diese Information kann je nach Fallkonstellation forensisch von großer Bedeutung sein [4, 16, 19].

**Abb. 9:**
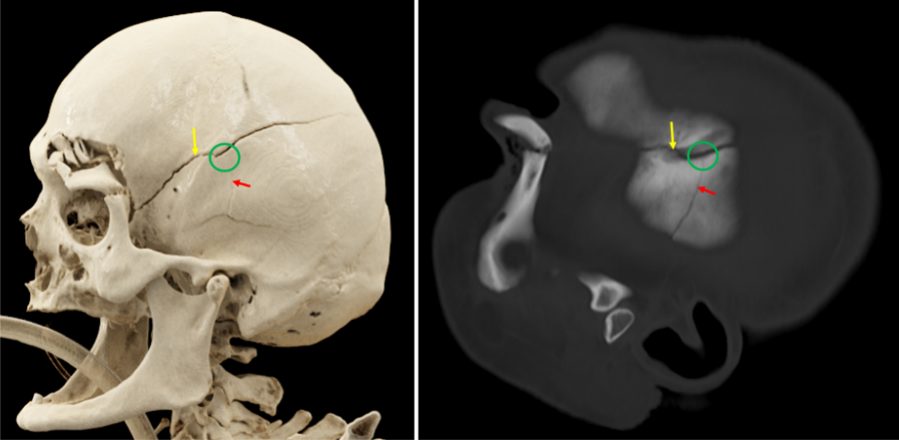
Postmortale Computertomographie (PMCT), Puppe-Regel: mehrfache stumpfe Gewalt gegen den Kopf mit Schädelfrakturen an der linken Schädelseite. Die später entstandene untere Bruchlinie (*roter Pfeil*) endet (*grüne Umkreisung*) an der früher entstandenen oberen Bruchlinie (*gelber Pfeil*)

Erwähnt sei noch die in der Rechtsmedizin verwendete und auch forensisch-radiologisch relevante Puppe-Regel die besagt, dass bei zeitlich aufeinanderfolgenden Schädelbrüchen die später entstandene Bruchlinie an der früher entstandenen endet. Es lassen sich somit bei mehreren Gewalteinwirkungen gegen den Kopf Aussagen über deren zeitliche Abfolge treffen ([4, 16, 19]; Abb. 9).

### Stumpfe Gewalt gegen den Rumpf

Forensisch relevante, stumpfe Rumpfverletzungen entstehen durch Druck- und Zugspannungen bei schweren Gewalteinwirkungen wie bspw. Verkehrsunfällen oder Stürzen aus der Höhe [4, 16, 19].

Bei Verletzungen der Thorax- und Lendenwirbelsäule kommt der PMCT besondere Bedeutung zu, da insbesondere Wirbelfrakturen dank der PMCT bereits präautoptisch gut zu erkennen sind und damit auch eine gezielte autoptische Präparation der betroffenen Region ermöglichen. Verletzungen der spinalen Achse sind forensisch bedeutend, da sie einen spinalen Schock auslösen und dadurch todesursächliche Relevanz haben können [4, 5, 11, 15, 22, 24].

Bei schwereren stumpfen Verletzungen der Thorax- oder Lendenwirbelsäule, etwa bei Translationsverletzungen oder bei Hyperflexionsverletzungen mit Berstungsfrakturen und intraspinalen Stempelfragmenten, sind Verletzungen des Myelons häufig bereits in der PMCT erkennbar. Die PMCT allein reicht jedoch nicht aus, um Rückenmarkverletzungen auszuschließen. Die PMMR wäre aufgrund des höheren Weichteilkontrastes grundsätzlich besser dafür geeignet, doch fehlen dazu verlässliche Studiendaten. In der PMCT oder PMMR sichtbare intraspinale Hämatome sind ebenfalls Hinweise auf eine Rückenmarkverletzung und sollten eine autoptische Untersuchung des Spinalkanals nach sich ziehen. Wie beim Schädel lassen sich auch bei der Wirbelsäule Frakturen teils besser und für medizinische Laien wie Juristen verständlicher darstellen als bei der Autopsie. Deswegen sollten die Bildgebungsbefunde bei rekonstruktiven Fragestellungen immer mitberücksichtigt werden.

In der PMCT sind Brustkorbverletzungen als Folge von mechanischen Reanimationsmaßnahmen relativ häufig zu beobachten , die streng genommen ebenfalls unter stumpfen Gewalteinwirkungen einzuordnen sind. Typischerweise finden sich hierbei beidseitige Rippenbrüche in den medioklavikulären Rippenanteilen, begleitet von einem Querbruch des Brustbeins. Forensisch herausfordernd kann dabei die Unterscheidung zwischen originär durch ein vorangegangenes stumpfes Trauma verursachten Rippenbrüchen oder agonal/postmortal durch Reanimationsmaßnahmen verursachten Rippenbrüchen sein. Gerade die unikortikalen anterolateraen Knickfrakturen, die im Rahmen einer Reanimation auftreten, sind radiologisch schwieriger zu erkennen als bei der Autopsie [23]. Mittels auf die Rippen gekippten multiplanaren Rekonstruktionen (MPR) sind sie jedoch klarer abgrenzbar [25].

Ebenso wie scharfe Gewalt kann auch schwere stumpfe Gewalt gegen den Brustkorb durch Verletzung von Atemwegen und Blutgefäßen zur Ausbildung eines Hämato- und/oder Pneumothorax führen, wobei insbesondere die Ausbildung eines Spannungspneumothorax mit Mediastinalverlagerung forensisch von Bedeutung ist, da dies todesursächliche Relevanz haben kann [4, 19]. Die PMCT ist hierbei in der Regel besser als die Autopsie geeignet, das eigentliche Ausmaß von Pneumothorax und Spannungskomponente darzustellen [5, 11, 15, 24]. Dasselbe gilt auch für das posttraumatische Pneumoperikard, welches häufiger nach Sturz aus großer Höhe aufzutreten scheint [10].

**Abb. 10:**
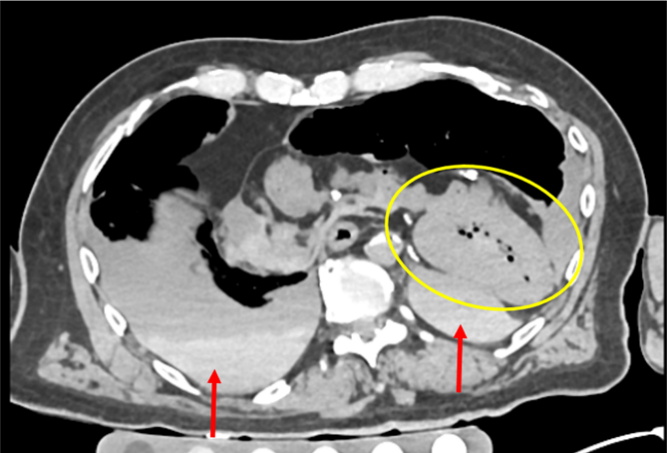
Postmortale Computertomographie (PMCT). Tödliche Herzruptur mit subtotalem Abriss der Herzbasis, Luxation des Herzens in die linke Brusthöhle (*gelbe Umkreisung*) und Blutung in die Brusthöhlen (*rote Pfeile*) bei Suizid als unangeschnallter PKW-Fahrer mit Frontalkollision

Durch massive stumpfe Brustkorbkompression kann es zu Prellungen und Quetschungen von Herz und Lungen kommen. Das Herz rupturiert dabei vor allem im Bereich der rechten Herzkammer, wobei die eigentliche Rupturstelle in der nativen PMCT in der Regel nicht zu erkennen ist (Abb. 10). Eine diagnostisch höhere Genauigkeit der konkreten Rupturstelle wäre mit einer postmortalen Angiographie oder einem PMMR des Herzens zu erwarten [9, 21]. Als indirekter Hinweis auf Rupturen des Herzens bei Vorliegen von stumpfer Gewalt gegen den Brustkorb kann jedoch auch in der nativen PMCT das Vorliegen eines Hämatoperikards gewertet werden.

**Abb. 11:**
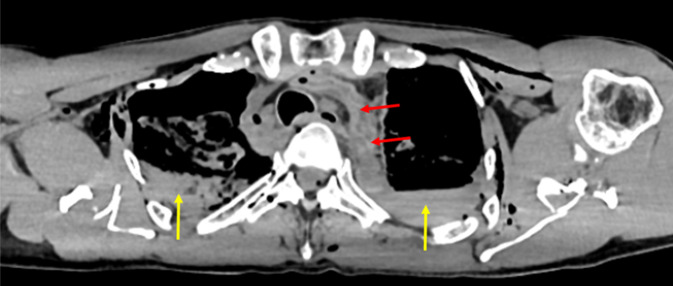
Postmortale Computertomographie (PMCT). Tödliche Aortenruptur im Bereich des Isthmus (*rote Pfeile*) und Blutverlust nach innen (*gelbe Pfeile*) bei Motorradunfall und Kollision mit Leitplanke

Eine häufige Todesursache bei stumpfer Gewalt gegen den Rumpf sind indirekte Aortenverletzungen nach Dezelerationstrauma. Diese treten klassischerweise am Aortenisthmus auf, wo die Aorta über das Ligamentum Botalli fixiert ist und auf vertikale bzw. horizontale Zugkräfte besonders vulnerabel ist [4, 16]. In der nativen PMCT kann der Verdacht auf eine solche Aortenverletzungen häufig nur aufgrund sekundärer Befunde wie eines ausgedehnten Hämatomediastinums, eines Hämatothorax und einer halbmondförmig kollabierten Aorta geäußert werden (Abb. 11). Zur eindeutigen radiologischen Diagnosestellung einer Aortenruptur wäre die postmortale CTA erforderlich [9].

Bei schweren stumpfen Gewalteinwirkungen gegen den Bauch sind Rupturen der inneren Organe wie Leber, Milz und Darm zu beobachten. Die eigentlichen Rupturstellen der Organe sind in der nativen PMCT üblicherweise nicht direkt oder nur schwer zu erkennen, so dass häufig lediglich freies Gas oder Blutansammlungen im Abdomen indirekt auf derartige Verletzungen hinweisen [6]. Bei der Leber hat sich gezeigt, dass fokal gefangenes Gas ein wichtiger Hinweis auf eine Leberlazeration darstellt [18].

In sehr seltenen Fällen kommt es nach stumpfer Gewalt zur traumatischen Ruptur der Bauchaorta, insbesondere bei vorbestehenden starken arteriosklerotischen oder aneurysmatischen Veränderungen. In der PMCT sollte daher bei Zeichen eines stumpfen Rumpftraumas und größeren abdominalen Blutansammlungen bei arteriosklerotisch veränderter Bauchaorta auch an deren Ruptur gedacht werden [4, 6].

Stumpfe Gewalt kann auch zu Nierenverletzungen und retroperitonealen Einblutungen führen, die in der PMCT oder PMMR meist besser darstellbar sind als bei der Autopsie. Knöcherne Beckenverletzungen können in der PMCT ebenfalls gut erkannt werden, sodass autoptische komplexe Beckenpräparationen oft entfallen können.

**Abb. 12:**
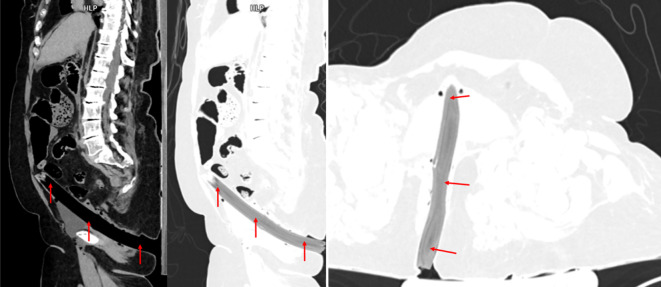
Postmortale Computertomographie (PMCT). Suizidaler Sturz aus der Höhe auf waldiges Terrain mit akzidenteller perianaler Pfählungsverletzung durch einen Holzast (*rote Pfeile*)

Sehr selten sind Pfählungsverletzungen mit stumpfen Gegenständen, die vor allem im Rahmen von Verkehrsunfällen oder Stürzen aus der Höhe zu beobachten sind und in der Regel todesursächliche Relevanz haben ([8]; Abb. 12).

### Extremitäten

**Abb. 13:**
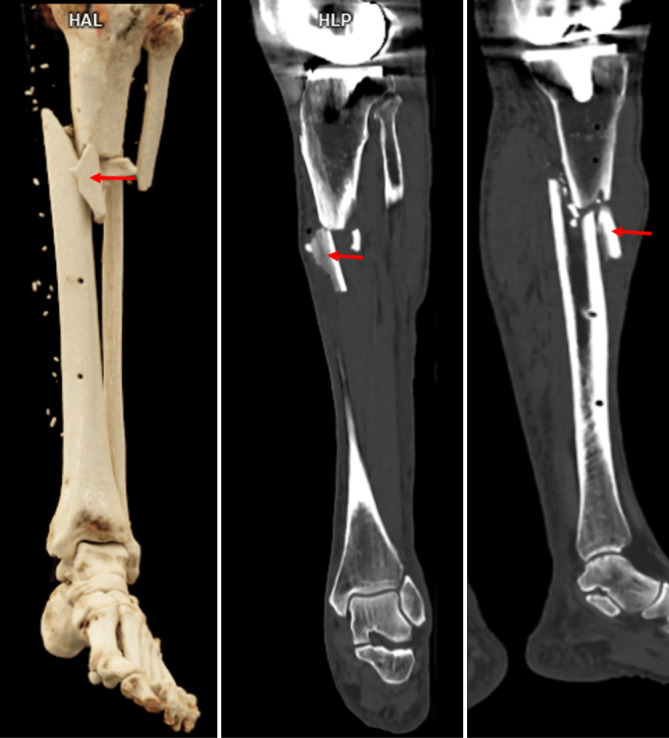
Postmortale Computertomographie (PMCT). Tödliche Kollision als Fußgänger mit einem Auto und Ausbildung eines Messerer-Keils (*rote Pfeile*) an der linken Tibia

Stumpfe Gewalteinwirkungen gegen die langen Röhrenknochen der Extremitäten führen durch Kompression und Biegung zu Frakturen, die je nach Art der Einwirkung zu unterschiedlichen Frakturmustern führen können. Bei Fußgänger-PKW-Kollisionen mit unklarem Geschehenshergang ist das Vorhandensein eines Keilbruchsystems (sog. Messerer-Keil) am Unterschenkel forensisch-rekonstruktiv von großer Bedeutung, da die Keilbasis den Ort der Gewalteinwirkung und die Keilspitze die Richtung der Gewalteinwirkung (bzw. des PKW) anzeigt ([3, 4, 13]; Abb. 13).

## Stumpfe Gewalt bei Lebenden (klinische Rechtsmedizin)

Zum Gebiet der klinischen Rechtsmedizin gehört auch die Dokumentation von Verletzungen, die durch körperliche Übergriffe aufgetreten sind. Bei Frauen und älteren Menschen erfolgen Übergriffe häufig im Rahmen von häuslicher Gewalt, bei (jüngeren) Männern meistens in Zusammenhang mit Auseinandersetzungen im öffentlichen Raum [1, 2, 7, 12, 17, 20].

Verletzungen, die durch stumpfe Gewalt entstehen, lassen sich grob in Angriffsverletzungen und Abwehrverletzungen einteilen [1]. Angriffsverletzungen werden durch direkte Faustschläge, Tritte oder Schläge mit stumpfen Gegenständen gegen den Kopf, den Rumpf oder die unteren Extremitäten verursacht. Abwehrverletzungen entstehen an den Händen und Unterarmen beim Versuch, Schläge gegen den Kopf abzuwehren.

Bei Gewaltbetroffenen, die sich nach einem körperlichen Übergriff in einer Notfallambulanz oder im Krankenhaus vorstellen, erfolgt die Indikation zur diagnostischen Bildgebung grundsätzlich aus klinisch-therapeutischen Gründen und nicht aus forensischen Überlegungen. Trotzdem können radiologische Untersuchungen von Gewaltbetroffenen sowie die entsprechenden Befundberichte für forensische Abklärungen herangezogen werden.

In der Klinik unterscheidet sich die Befundung radiologischer Untersuchungen bei Gewaltbetroffenen nicht von denen anderer Patientinnen und Patienten. Der radiologische Befundbericht sollte auch in diesen Fällen eine objektive Beschreibung sowie eine klinische – und keine forensische – Interpretation der Befunde enthalten.

**Abb. 14:**
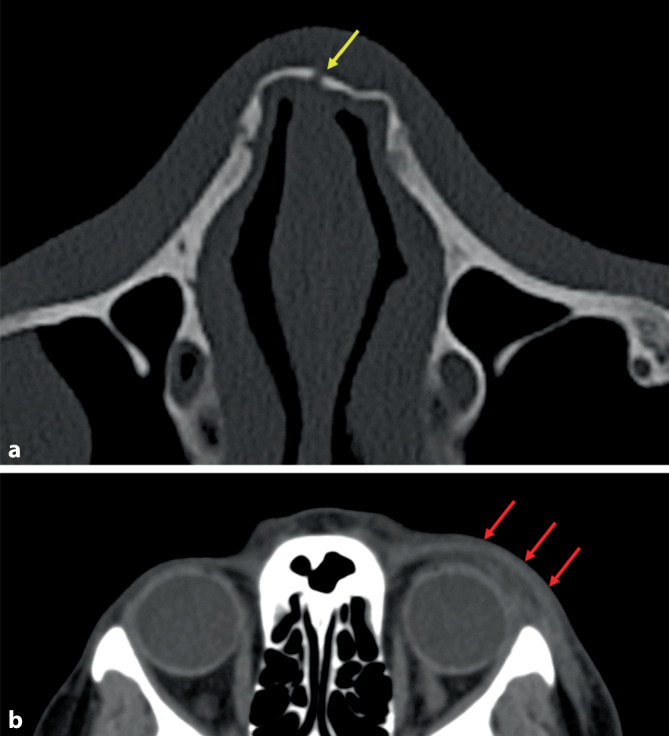
Klinische Computertomograhphie (CT), Nasenbeinfraktur (**a**, *gelber Pfeil*) und Monokelhämatom links (**b**, *rote Pfeile*) nach Faustschlägen gegen das Gesicht (unterschiedliche Patienten)

Häufige computertomographisch fassbare Angriffsverletzungen bei Betroffenen von häuslicher Gewalt sind das periorbitale Hämatom – meist links bei rechtshändigem Angreifer – und die Nasenbeinfraktur ([1, 7]; Abb. 14). Obwohl diese Befunde für nichtakzidentelle Verletzungen nicht spezifisch sind, gilt die Kombination aus akutem periorbitalem Hämatom und einer alten Nasenbeinfraktur in derselben Schädel-CT als starkes Indiz für wiederholte häusliche Gewalt [1].

**Abb. 15:**
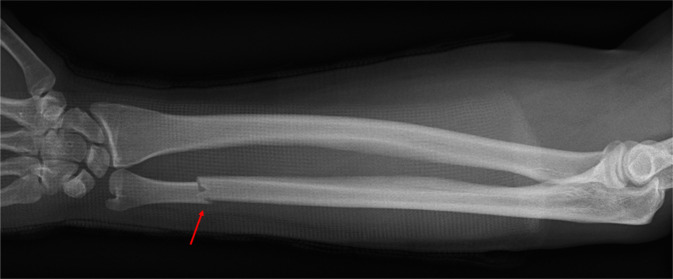
Röntgenaufnahme einer durch Selbstunfall verursachten Ulnafraktur

Zu den typischen radiologisch fassbaren Abwehrverletzungen gehören die isolierte Ulnaschaftfraktur sowie ulnarseitige Frakturen der Metakarpalknochen und Finger, insbesondere von Os metacarpale V und Digitus V und IV, wobei diese Verletzungen nicht pathognomonisch für körperliche Übergriffe sind: Auch die isolierte Ulnaschaftfraktur, die als klassische Abwehrverletzung gilt, kann bei Unfallereignissen auftreten ([12]; Abb. 15).

### Infobox Häusliche Gewalt

Häusliche Gewalt ist länderübergreifend ein großes gesellschaftliches Problem, von dem vor allem Frauen, seltener auch Männer betroffen sind. Die Gewaltausübung kann physisch, sexuell und psychisch sein oder eine Kombination dieser Formen umfassen. Bei physischer Misshandlung handelt es sich zumeist um stumpfe Gewalteinwirkung, häufig auch mit Angriffen gegen den Hals. Häusliche Gewalt hat eine hohe Dunkelziffer und wird oft ärztlich nicht erkannt.

Radiologinnen und Radiologen kann bei der Erkennung physischer häuslicher Gewalt große Bedeutung zukommen, da sie Zugriff auf die radiologische Patienten- bzw. Befundhistorie haben und mit Kenntnis der typischen radiologischen Befundmuster bei häuslicher Gewalt Hinweise auf diese erkennen können.

Radiologische Hinweise auf häusliche Gewalt bzw. nichtakzidentelle Verletzungen:

- *Abwehr- und Angriffsverletzungen:* Abwehrverletzungen finden sich häufig medialseitig an den oberen Extremitäten, insbesondere an Ulna, Mittelhand und Fingern. Angriffsverletzungen treten typischerweise an Kopf bzw. Gesicht auf, insbesondere Prellungen mit Weichteilschwellungen und Hämatomen, oft auch Nasenbeinfrakturen und linksseitige Orbitafrakturen sowie dorsale Rippenfrakturen (durch Stoßen). Nur ein Teil von klinisch erkennbaren Abwehr- und Angriffsverletzungen ist radiologisch fassbar.
- *Mehrzeitige Verletzungen:* Das Vorliegen von akuten und älteren Verletzungen – wie die bereits erwähnte Kombination aus periorbitalem Hämatom und alter Nasenbeinfraktur – ist grundsätzlich verdächtig für wiederholte, nichtakzidentelle Verletzungen. Auch bei Rippenfrakturen in unterschiedlichem Heilungszustand ist an nichtakzidentelle Verletzungen zu denken.
- *Entstehungsmechanismus nicht plausibel:* Frakturen, die durch den geltend gemachten Entstehungsmechanismus nicht plausibel erklärt werden können, gelten als Warnsignal für nichtakzidentelle Verletzungen.
- *Rolle der Radiologie bei Verdacht auf häusliche Gewalt:* Radiologinnen und Radiologen sollten Verdachtsfälle nichtakzidenteller Verletzungen mit der zuweisenden klinischen Ärzteschaft besprechen, um dem Behandlungsteam die Gelegenheit zu geben, dieses Thema in einem geeigneten Umfeld mit den Betroffenen anzusprechen. Ein unmittelbarer Beizug der Strafverfolgungsbehörden bzw. Polizei sollte auf Wunsch des Opfers oder bei unmittelbar drohender Gefahr für Leib und Leben, etwa dem Vorliegen lebensbedrohlicher Verletzungen oder bekannter Tötungsdrohungen des Partners, erfolgen.

## Fazit für die Praxis

- Verletzungsbedingte Folgen stumpfer Gewalt sind in der forensisch-radiologischen Diagnostik häufig zu beobachten, vor allem im Rahmen von Unfällen und Suiziden, seltener bei Tötungsdelikten.
- Die Methode der Wahl zur radiologischen Darstellung von stumpfer Gewalt bei Verstorbenen ist die native postmortale Computertomographie (PMCT).
- Grundsätzlich unterscheiden sich Folgen stumpfer Gewalt radiologisch in der PMCT nicht wesentlich vom Lebenden.
- Die postmortale Magnetresonanztomographie (PMMR) ist im kürzeren postmortalen Intervall sehr gut zur Darstellung von stumpfen Weichteilverletzungen geeignet.
- Bei Lebenden mit Folgen stumpfer Gewalt erfolgt die Indikation zur Bildgebung primär aus klinisch-diagnostischen Gründen.
- Häufige Indikationen sind häusliche Gewalt, Gewalt an älteren Personen sowie Auseinandersetzungen im öffentlichen Raum, wobei die radiologischen Untersuchungen einer forensischen Begutachtung unterzogen werden können.
